# Case Report: Uncommon Association of *ITGB4* and *KRT10* Gene Mutation in a Case of Epidermolysis Bullosa With Pyloric Atresia and Aplasia Cutis Congenita

**DOI:** 10.3389/fgene.2021.641977

**Published:** 2021-07-08

**Authors:** Melinda Matyas, Diana Miclea, Gabriela Zaharie

**Affiliations:** ^1^Neonatology, Iuliu Haţieganu University of Medicine and Pharmacy, Cluj-Napoca, Romania; ^2^Genetics, Iuliu Haţieganu University of Medicine and Pharmacy, Cluj-Napoca, Romania

**Keywords:** epidermolysis bullosa, aplasia cutis, newborn, pyloric atresia, DNA sequencing

## Abstract

**Background:** Epidermolysis bullosa is a rare form of genodermatosis produced by different gene mutations. The junctional form of the disease (JEB-PA) can associate pyloric atresia, renal abnormalities, and aplasia cutis congenita.

**Case Description:** A case of a male preterm newborn with suspicion of digestive tube malformation at fetal ultrasound and who was born by cesarian section. At birth, he presented extensive cutaneous aplasia on the lower limbs and bilaterally under ears; outer ear agenesis; nasal septum hypoplasia; micrognathia; multiple blisters on the face, trunk, and limbs; lower limb deformities and absence of toe nails. Pathological examination following a surgical procedure with unfavorable outcome showed pyloric atresia, junctional form of epidermolysis bullosa and aplasia cutis congenita. Homozygous variants in two genes were identified: c.3111+1G>A in *ITGB4* (class 5) and c.1498G>T in *KRT10* (class 3).

**Conclusion:** The particularity of our case is the novel finding of a coincidental occurrence in the context of consaguinity of two mutations in the *ITGB4* and *KRT10* genes, and clinical characteristics of epidermolysis bullosa.

## Introduction

Epidermolysis bullosa is a group of rare genetic disorders characterized by skin fragility and mechanically induced blistering. This group of genetic disorders is clinically heterogeneous, includes a broad spectrum of severity and more than 30 clinical subtypes (Has et al., 2020a).

Junctional epidermolysis bullosa has different clinical subtypes. The clinical form with pyloric atresia (JEB-PA) is characterized by fragility of the skin and mucous membranes, manifested by blistering with little or no trauma; and congenital pyloric atresia. Additional manifestations found in patients with JEB-PA include congenital localized absence of skin (aplasia cutis congenita) affecting the extremities and/or head; nail dystrophy; scarring alopecia; hypotrichosis; contractures; and dilated cardiomyopathy. The course of JEB-PA is usually severe and often lethal in the neonatal period (Carmi et al., 1982; Maman et al., 1998; Birnbaum et al., 2008), with the mortality of cases reaching 80% (Pfendner and Lucky, 1993; Hon et al., 2015).

The keratin 10 (*KRT10*) gene mutation is associated with keratinopathic ichthyoses, a form of hyperkeratotic disorders with skin fragility. The skin cleavage level is intraepidermal, and the inheritance can be autosomal recessive or autosomal dominant (Richards et al., 2015). We are presenting the case of a newborn with suspicion of digestive tube malformation from fetal stage. The case is unique as it shows associated *KRT10* and *ITGB4* gene mutations, an association that has not been described previously.

## Patient Information

This is the case of a male preterm newborn with a gestational age of 32 weeks. The mother's history included a healthy child and an extremely premature infant deceased at birth. The current pregnancy was followed up and the birth was by cesarean section due to a suspicion of digestive tub malformation—enlarged stomach at fetal ultrasound. The fetal ultrasound also revealed hypertelorism, facial abnormalities, short limbs. The parents are clinically healthy, affirmatively, the parents are second degree cousins.

## Clinical Findings

After delivery, the child was admitted to the neonatal intensive care unit where he was noted to present extensive cutaneous aplasia on the lower limbs and bilaterally under ears; outer ear agenesis; nasal septum hypoplasia; micrognathia; multiple blisters on the face, trunk and limbs; lower limb deformities and absence of toe nails.

## Diagnostic Assessment

A head ultrasound showed enlarged interhemispheric and subarachnoid space; an abdominal ultrasound revealed important hepatomegaly and significantly distended stomach occupying the abdominal cavity. Both ultrasound examinations were performed with difficulty because of the extensive cutaneous lesions.

The patient was transferred to the service of pediatric surgery, where he underwent a surgical procedure. He died at the age of 5 days. Pathological examination showed pyloric atresia, junctional form of epidermolysis bullosa and aplasia cutis congenita.

After obtaining informed consent, genomic DNA was extracted from the patient's peripheral blood samples using standard methods and analyzed using next generation sequencing (NGS). Exome sequencing was done using the Agilent SureSelect Human All Exon V6 capture kit on an Illumina HiSeq PE150 platform and we obtained a minimum of 50X human WES per sample. The genome used was hg38 and variant annotation was done using Annovar.

The interpretation of exome data showed two different homozygous null variants, one a mutation affecting a splice site in *ITGB4* gene - NM_000213:c.3111+1G>A, and the other, a non-sense mutation in *KRT10* gene - NM_000421:c.1498G>T.

The *ITGB4* (c.3111+1G>A) gene is affected by a homozygous variant which induces a splice site mutation in exon 26. It is a rare variant, not found in the gnomAD database. This variant is predicted as pathogenic by several prediction platform (BayesDel_addAF, EIGEN, FATHMM-MKL, MutationTaster, and scSNV-Splicing). Impairment of the *ITGB4* gene results in the following clinical phenotype: junctional epidermolysis bullosa with pyloric atresia (autosomal recessive—AR transmission). The patient's phenotype is consistent with this description. According to ACMG criteria, this variant is classified as class 5 variant (pathogenic) (PVS1 as null variant, PM2 due to its rarity and PP3 due to computational prediction).

**Figure 1:**
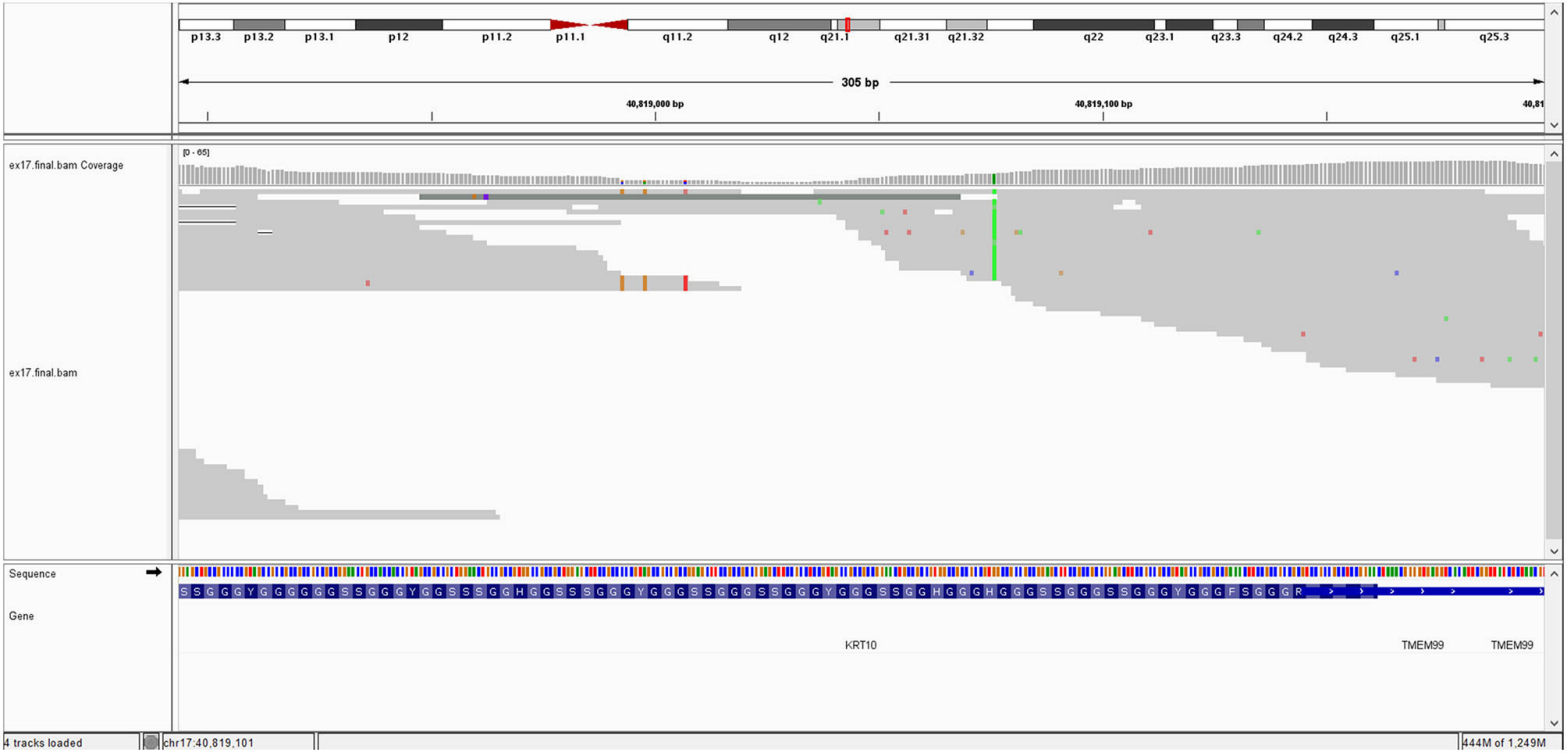
ITGB4 gene mutation.

The image of mutation is show in Figure 1.

**Figure 2:**
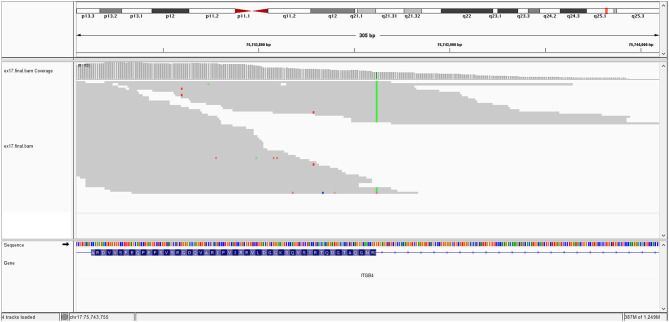
KRT10 gene mutation.

The *KRT10* gene homozygous variant induces a stop codon in exon 7; it has a frequency of <0.01% in the gnomAD databases. *In silico* prediction is heterogenous in different computational platforms, some indicate it as pathogenic (Mutation Taster, BayesDel) other benign (EIGEN) or neutral (FATHMM, LRT). However, a counter argument for the pathogenicity could be the description of benign homozygous frameshift variant (such as *KRT10* (NM_000421.5):c.1459_1460insGGCGGCGGCT (p.His487ArgfsTer97), hg38), located upstream, in the same exon as the variant described at our patient. Thus, according to these data and based on American College of Medical Genetics (ACMG) criteria (PVS1 and PM2 criteria), even if it is responsible of a stop codon (null variant), we interpret it as class 3 variant (variant of uncertain significance) (Richards et al., 2015). Pathogenic *KRT10* variants are associated with congenital reticular ichthyosiform erythroderma (also known as ichtyosis with confetti) (AD transmission) (Guerra et al., 2015); Aaru disease and cyclic ichthyosis (AD transmission) or with keratynophatic ichtyosis (AD or AR transmission). The targeted protein is keratin 10 in AR form and keratin 1, 2, and 10 in AD form (Sybert et al., 1999; Guerra et al., 2015). The mutation of KRT10 gene is presented by Figure 2.

The exome data indicated a high rate of homozygous variants (105,640 variants were homozygous from the total of 180,380 variants, or 58%). The parents were healthy, without dermal clinical changes. The parental DNA was not available to perform the genetic testing for the family trios. Genetic counseling was performed for the parents, including an a priori recurrence risk of 25% of disease transmission by *ITGB4* homozygous variants to other descendants, also with the recommendation to present to geneticist as soon as they are planning a new pregnancy or as soon as the pregnancy is diagnosed, to discuss all the options for prenatal diagnosis.

## Discussion

Epidermolysis bullosa types are defined based on the location of the disruption to the skin structure. Based on the ultrastructural level of skin cleavage there are 4 types of epidermolysis bullosa, caused by pathogenic variants in 18 different genes: epidermolysis bullosa simplex (EBS), junctional epidermolysis bullosa (JEB), dystrophic epidermolysis bullosa (DEB), and Kindler EB (Fine et al., 2008, 2014; Has et al., 2020b).

**Figure 3:**
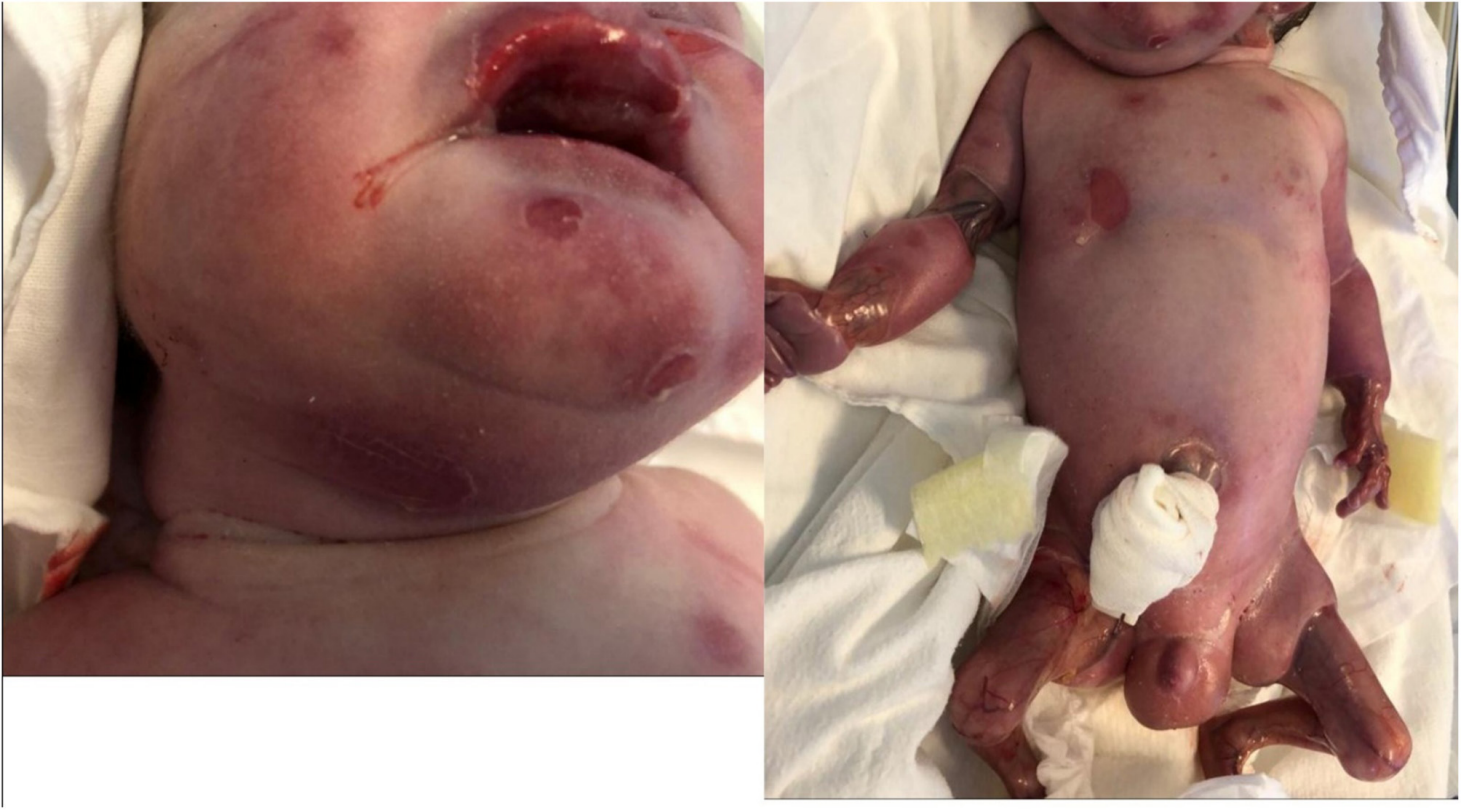
Bullous lesions on face and trunk. Small zones of aplasia cutis on upper limb.

Our patient had multiple blisters on the face, trunk and limbs and few blisters were on the oral mucosae (Figure 3). Blistering of the skin are the most common finding in epidermolysis bullosa. The blisters can develop as result of birth trauma. The presented case had limited exposure to such a trauma as birth was by cesarean section. Blisters can also occur on the mucosae: the oral mucosa, the digestive tract mucosa, as well as the conjunctiva. The plane of cleavage is localized in lamina lucida of the cutaneous membrane zone (Has et al., 2020a).

Pyloric atresia is most frequently associated with the JEB form, although it has been described in other types of epydermolisis bullosa as well (Pfendner and Lucky, 1993). Classification of JEB is based on severity and consists of JEB severe (former generalized severe, Herlitz JEB) and JEB intermediate (previously known as intermediate severe, non–Herlitz JEB). In JEB severe, hemidesmosomes are hypoplastic and reduced in number and the anchoring filaments are significantly reduced or absent. In the JEB intermediate form, the hemidesmosomes may be reduced or hypoplastic, and the anchoring filaments may also be reduced (Shinkuma et al., 2011; Kelly-Mancuso et al., 2014; Richards et al., 2015). In the severe form of the disease, a high rate of early lethality in the first month of life is reported. Pyloric atresia may be suspected during gestation as a result of oligohydramnios, with or without elevated alpha-fetoprotein and acetylcholinesterase levels, and echogenic material in the amniotic fluid. The parents of an affected child are usually obligate heterozygotes (i.e., carriers of one *COL17A1, ITGB4, LAMA3, LAMB3*, or *LAMC2* pathogenic variant), and because germline mosaicism and uniparental isodisomy have been reported, the carrier status of parents would need to be confirmed through molecular genetic testing (Cserhalmi-Friedman et al., 2002; Fassihi et al., 2005). In our case, the digestive tube malformation was suspected due to the enlarged stomach found on fetal ultrasound. The abdominal ultrasound done after birth revealed an enlarged stomach that occupied the abdominal cavity. The diagnosis of pyloric atresia was sustained later by the surgery and confirmed by pathological exam after the death of the patient.

**Figure 4:**
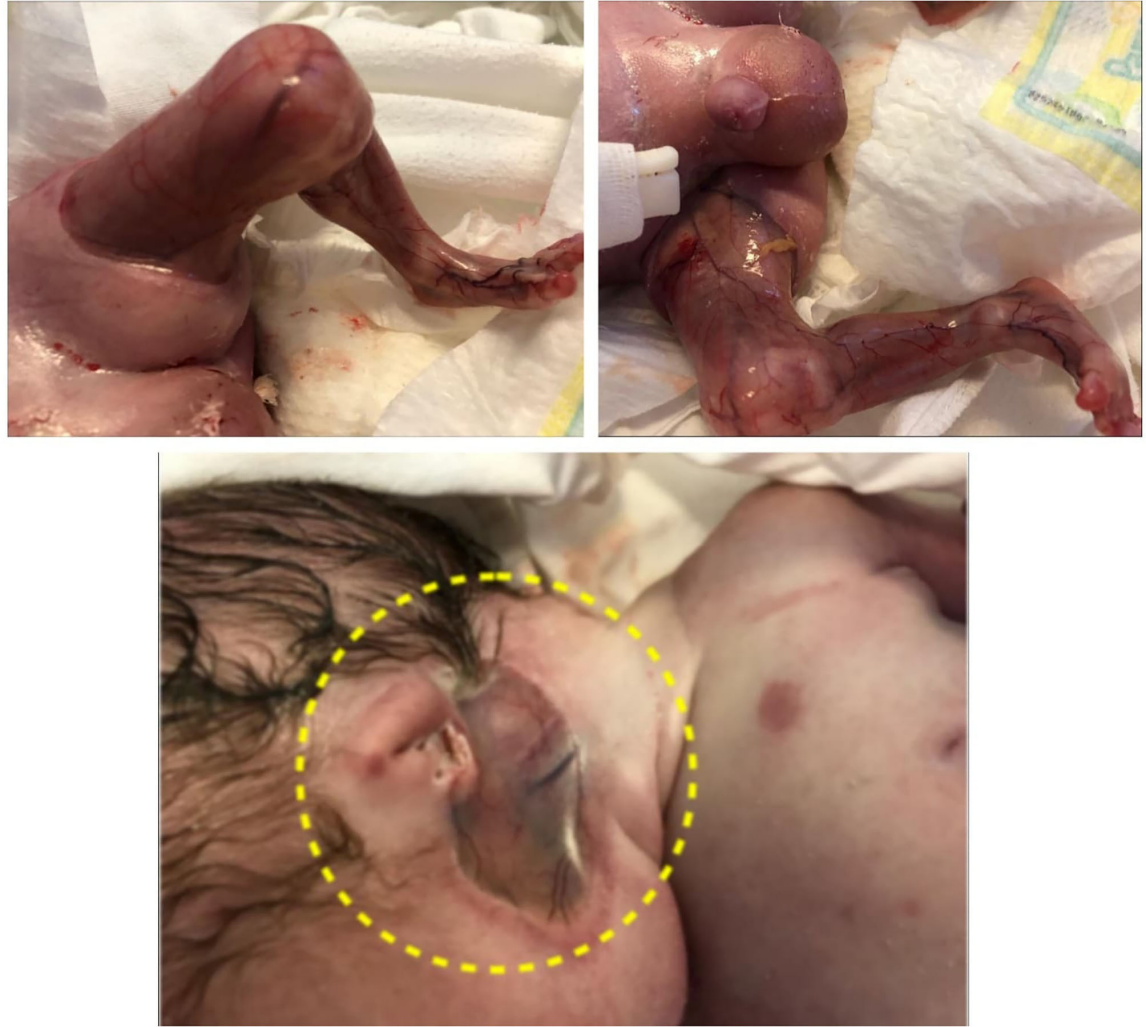
Aplasia cutis congenital of lower limbs, absence of toenail. Outer ears absence.

Extensive aplasia cutis areas are also described in JEB-PA. Malformation or lack of finger and toenails can be present, with pseudo-syndactyly, defined as the partial or complete loss of web spaces between any digits of the hands or feet, being a rare. Joint deformations and contractures may appear, limiting mobility. The reported case presented extended areas of aplasia cutis on the lower limbs and under the ears. Also lack of toenails was present. On the limbs, mainly on lower limbs, contractures with limited mobility were present. It was impossible to extend the lower limbs due to the join deformation and contractures that were present (Figure 4).

The elements characterizing JEB-PA: limb deformities, strictures, scars, the absence of toenails and the absence of outer ears were present in the reported case (Chiaverini et al., 2014; Diociaiuti et al., 2016). Cutaneous aplasia can be associated with all forms of epidermolysis bullosa and involves the same ultrastructural changes, Location in the lower limbs is predominant (Kanzler et al., 1992).

JEB is less common than the DEB or EBS type. The clinical subtypes can associate not just pyloric atresia, but also interstitial lung disease and nephrotic syndrome. In the form of JEB with PA, the targeted protein is integrin α6β4, while in the subtype associating lung disease, the targeted protein is the integrin α3 subunit.

The particularity of our patient resides in the fact that genetic testing found two substitutions, one in the *ITGB4* gene and another in the *KRT10* gene.

In the *ITGB4*, a nucleotide following the last nucleotide of exon 26 (the first nucleotide of the intron) has a substitution of guanine with adenine: c.3111+1G>A. This occurs exactly at the splice site which it suppresses and because of this, an intron remains in the gene, in the non-coded sequence. Mutations of *ITGB4* are most often involved in JEB-PA (80% of cases). This is a novel variant that has not been described previously in population databases, such as gnomAD. We consider it as pathogenic based on ACMG criteria and the consistent phenotype of our patient. The involvement of the *ITGB4* gene is responsible for a phenotype that largely overlaps our patient's phenotype, junctional epidermolysis bullosa with pyloric atresia. In the *KRT10 gene*, a substitution of guanine with thymine in exon 7 (of 8), induces a stop codon (c.1498G>T; p.Gly500^*^). We interpret it as uncertain variant, based on ACMG criteria, with an argument for pathogenicity due to non-sense mutation, but with some reserve due to the possibility of non-pathogen null variants described in this gene.

It is known that junctional epidermolysis bullosa with pyloric atresia (*ITGB4*) is an autosomal recessive disorder, but the involvement of the same gene can also be responsible for other disorders, with dominant inheritance, such as epidermolysis bullosa of the hands and feet, which is excluded here (both phenotypically and through the genotypic effect) (Iacovacci et al., 2003). On the other hand, homozygosity seen in our patient involves the presence of a heterozygous status in each of the two parents, but none of the two is affected by a cutaneous phenotype, which rather indicates that these variants are mainly responsible of autosomal recessive phenotype.

At the same time, the presence of a null allele in the *ITGB4* gene is responsible for a form with greater severity that is frequently lethal, compared to other types of mutations such as missense mutations. The *ITGB4* gene is also interesting, both in association with developmental abnormalities, and in association with cancer pathology, in the case of somatic mutations(Li et al., 2019; Ruan et al., 2020; Sung et al., 2020).

This genetic diagnosis also brought into discussion the provision of adequate genetic counseling, given the parents' consanguinity. The risk of recurrence in a future offspring taken into consideration for genetic counseling purposes, primarily involved the homozygous *ITGB4* variant, noted as a class 5 variant in this patient. The *KRT10* variant, an uncertain variant, was also brought into discussion. Considering the parental consanguinity, indicated by exome analysis as a close degree of kinship, the possibility of transmission to a new offspring of other homozygous variants, responsible for other recessive diseases undetected in the index case should be taken into account. For a future pregnancy, considering the increased risk of recurrence, we recommended prenatal diagnosis with the sequencing of the two genes, and if possible, exome evaluation, to determine other homozygous variants possibly associated with other recessive diseases, in the context of consanguinity.

## Patient Perspective

The particularity of our case resides in the coincidental occurrence of homozygous variants of the *ITGB4 gene* (clearly interpreted as pathogenic) and *KRT10 gene*. A small number of JEB–PA with *IGTB4* have been associated with a survival of more than 1 year (Schumann et al., 2013; Ko et al., 2018), however the evolution of the presented case was unfavorable. Early surgery was required to resolve the digestive malformation. Postoperatively, multiple organ failure developed, causing progressive deterioration of the patient's status and ultimately, his death. The pathological examination confirmed the diagnosis of pyloric atresia. The mucosa was completely absent; submucosal calcium deposits and significant interstitial fibrosis between the muscle fibers were found. JEB-PA is often lethal, even when the gastric malformation is surgically repaired (Ko et al., 2018).

## Data Availability Statement

The datasets presented in this study can be found in online repositories. The names of the repository/repositories and accession number(s) can be found at: https://www.ebi.ac.uk/ena/browser/view/PRJEB44205, European Nucleotide Archive, Study no. ERP128229.

## Ethics Statement

Written informed consent was obtained from the individual(s), and minor(s)' legal guardian/next of kin, for the publication of any potentially identifiable images or data included in this article.

## Author Contributions

MM: conceptualization, data curation, and writing—original draft preparation. DM: genetic test preparation and interpretation. GZ: reviewing and editing. All authors contributed to the article and approved the submitted version.

## Conflict of Interest

The authors declare that the research was conducted in the absence of any commercial or financial relationships that could be construed as a potential conflict of interest.
